# Correlation of Preoperative Renal Insufficiency With Mortality and Morbidity After Aortic Valve Replacement: A Propensity Score Matching Analysis

**DOI:** 10.1097/MD.0000000000002576

**Published:** 2016-03-07

**Authors:** Chun-Yu Lin, Feng-Chun Tsai, Yung-Chang Chen, Hsiu-An Lee, Shao-Wei Chen, Kuo-Sheng Liu, Pyng-Jing Lin

**Affiliations:** From the Department of Cardiothoracic and Vascular Surgery Chang Gung Memorial Hospital (C-YL, F-CT, H-AL, S-WC, K-SL, P-JL), and Department of Nephrology, Chang Gung University College of Medicine, Taoyuan, Taiwan (Y-CC).

## Abstract

Preoperative end-stage renal disease carries a high mortality and morbidity risk after aortic valve replacement (AVR), but the effect of renal insufficiency remains to be clarified. Through propensity score analysis, we compared the preoperative demographics, perioperative profiles, and outcomes between patients with and without renal insufficiency.

From August 2005 to November 2014, 770 adult patients underwent AVR in a single institution. Patients were classified according to their estimated glomerular infiltration rate (eGFR) as renal insufficiency (eGFR: 30–89 mL/min/1.73 m^2^) or normal (eGFR, ≥90 mL/min/1.73 m^2^). Propensity scoring was performed with a 1:1 ratio, resulting in a matched cohort of 88 patients per group.

Demographics, comorbidities, and surgical procedures were well balanced between the 2 groups, except for diabetes mellitus and eGFR. Patients with renal insufficiency had higher in-hospital mortality (19.3% versus 3.4%, *P* < 0.001), a greater need for postoperative hemodialysis (14.8% versus 3.1%, *P* = 0.009), and prolonged intubation times (>72 hour; 25% versus 9.1%, *P* = .008), intensive care unit stays (8.9 ± 9.9 versus 4.9 ± 7.5 days, *P* = .046), and hospital stays (35.3 ± 31.7 versus 24.1 ± 20.3 days, *P* = .008), compared with those with normal renal function. Multivariate analysis confirmed that preoperative renal insufficiency was an in-hospital mortality predictor (odds ratio, 2.33; 95% confidence interval, 1.343–4.043; *P* = .003), as were prolonged cardiopulmonary bypass time, intraaortic balloon pump support, and postoperative hemodialysis. The 1-year survival significantly differed between the 2 groups including (normal 87.5% versus renal insufficiency 67.9%, *P* < .001) or excluding in-hospital mortality (normal 90.7% versus renal insufficiency 82.1%, *P* = .05).

Patients with preoperative renal insufficiency who underwent AVR had higher in-hospital mortality rates and increased morbidities, especially those associated with hemodynamic instabilities requiring intraaortic balloon pump support or hemodialysis. Earlier surgical intervention for severe aortic valve disease should be considered in patients who show deteriorating renal function during follow-up.

## INTRODUCTION

Hemodialysis-dependent patients with preoperative end-stage renal disease (ESRD) have an increased risk of morbidity and mortality after cardiac valvular surgeries such as aortic valve replacement (AVR).^1,2^ The rates of in-hospital mortality are high (6.8–17.3%) in these patients, and they experience more postoperative complications and prolonged hospitalization than those with normal renal function.^3,4^ To determine the effect of renal insufficiency, the preoperative demographics, perioperative profiles, and outcomes of patients with or without renal insufficiency, treated by AVR in a single institution, were compared using propensity score analysis.

## PATIENTS AND METHODS

This retrospective study was conducted with the approval of the Institutional Ethics Committee (No.104–7436B). The need for individual patient informed consent was waived.

Between August 2005 and November 2014, 846 consecutive patients underwent AVR in a single institution. After excluding those who were <18 years old, who had combined aortic surgery, or who underwent regular hemodialysis before the surgery, 770 patients were enrolled. Individual estimated glomerular filtration rates (eGFRs) were calculated using the modification of diet in renal disease (MDRD) equation,^5^ and patients were grouped into the renal insufficiency (eGFR 30–89 mL/min/1.73 m^2^; n = 100, 12.9%) or normal (eGFR ≥90 mL/min/1.73 m^2^; n = 670, 87.1%) groups. To reduce the effects of selection bias and potential confounding factors, a propensity score analysis was conducted.^6^ The propensity score for each patient was calculated by binary logistic regression including all the variables listed in Table 1. The propensity score was used to match patients with and without renal dysfunction in a 1:1 ratio, resulting in 88 matched pairs in whom postoperative outcomes were assessed. Preoperative demographics, associated comorbidities, surgical procedures, and hospital recoveries were compared between the groups. In-hospital mortality was defined as death occurring during hospitalization.

**TABLE 1:**
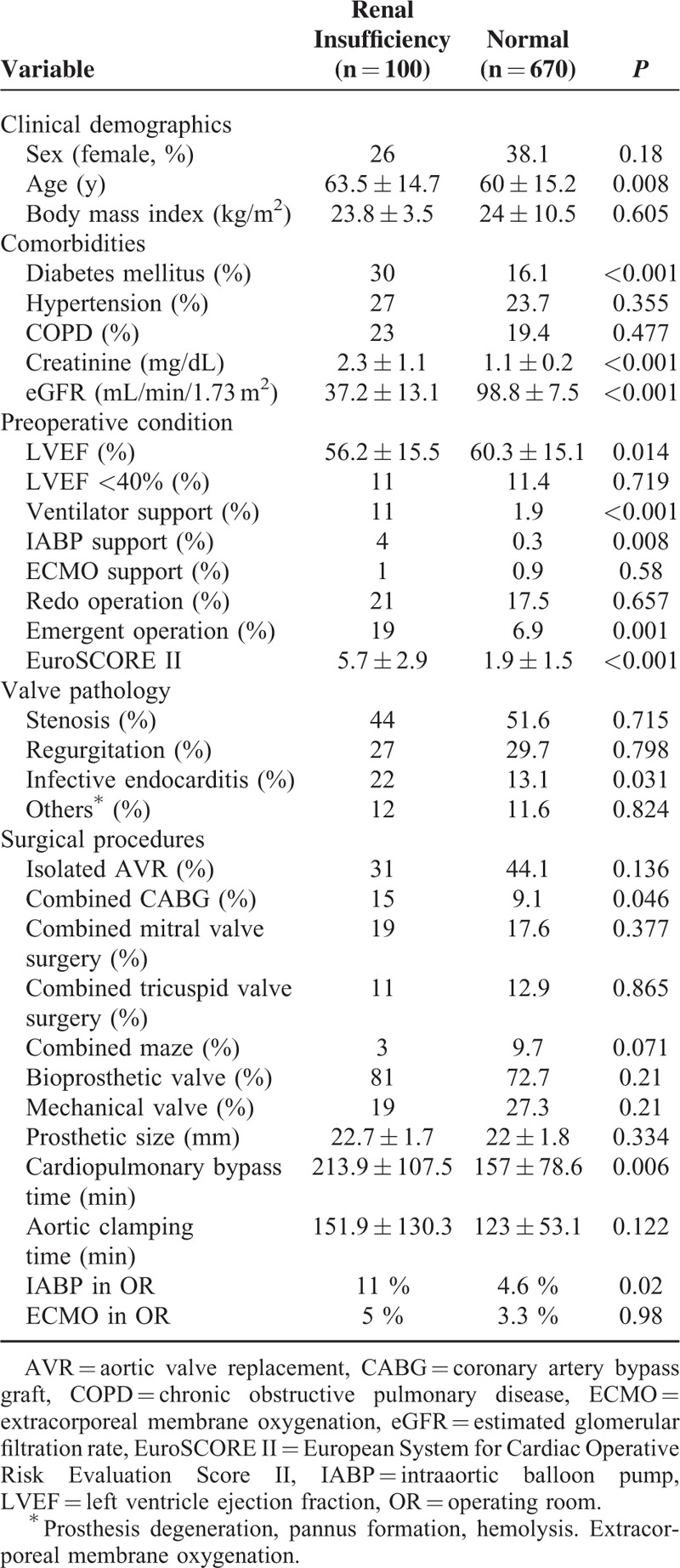
Comparisons of Clinical Demographics, Comorbidities, Preoperative Condition, and Surgical Procedures Between the Renal Insufficiency and Normal Groups

Emergent operations included those in patients with unstable hemodynamics requiring a high dose of inotropes, endotracheal intubation, or mechanical circulatory support, including intraaortic balloon pump (IABP) or extracorporeal membrane oxygenation (ECMO) before surgery. Following a preoperative discussion, the prosthesis type was selected at the discretion of the primary surgeon and by patient choice. We evaluated the severity of acute kidney injury within 48 hours after AVR, as determined from the definition of Acute Kidney Injury Network (AKIN) criteria. Acute kidney injury was considered when serum creatinine level increased over 1.5 × of the baseline or when urine output decreased to less than 0.5 mL/kg/h for over 6 hours.^7^ Besides, the Confusion Assessment Method for the intensive care unit (CAM-ICU) was applied to evaluate the status of delirium every 8 hour after admission to the ICU. Once delirium was suspected using the questionnaire, a psychiatrist was consulted to confirm the type of delirium: hyperactive, hypoactive, or mixed type.^8^

Statistical analyses were performed using SPSS for Windows (Version 22.0, SPSS, Chicago, IL). Data are presented as means ± standard deviation for the numerical variables, and as percentages for the categorical data. For all analyses, statistical significance was set at *P* < 0.05. Univariate analyses were performed using the independent *t* test, Mann–Whitney *U* test, χ^2^ test, or Fisher exact test to compare clinical demographics and postoperative complications. Significant univariables for in-hospital mortality (*P* < 0.05) were dichotomized based on cutoff values, which were determined by receiver operating characteristic curve analysis. These dichotomized risk factors were tested by multivariate logistic regression analysis, the Hosmer–Lemeshow test, and area under receiver operating characteristic curve (AUROC) analysis to create a prediction model of in-hospital mortality.^9^ Covariates for regression analysis included preoperative renal insufficiency, IABP or ECMO support in the operating room, postoperative hemodialysis, re-exploration for bleeding, stroke, prolonged intubation (>72 hour), ECMO support in the intensive care unit (ICU), ICU readmission, prolonged cardiopulmonary bypass (CPB) time (>149 minute), and ICU stay interval. Kaplan–Meier analysis was used to construct cumulative survival curves that were compared using the log-rank test.

## RESULTS

### Patient Characteristics and Propensity Matching

The clinical demographics, preoperative conditions, and surgical procedures were compared between the 2 groups as illustrated in Table 1. Other than having a significantly lower eGFR and higher serum creatinine level (*P* < .001), patients in the renal insufficiency were significantly older (*P* = .008) and had a higher prevalence of diabetes mellitus (DM) (*P* < 0.001) compared with those in the normal group. In terms of preoperative condition, patients with renal insufficiency had significantly more critical conditions, including worse left ventricular ejection fraction (*P* = 0.014), an increased prevalence of ventilator use (*P* < 0.001), the need for IABP support (*P* = 0.008), and the incidence of emergent operations (*P* = 0.001). The mean European System for Cardiac Operative Risk Evaluation Score II (EuroSCORE II) was significantly higher in patients with renal insufficiency compared with that in the normal group.

In terms of surgical variables, patients with renal insufficiency had a higher incidence of combined procedures, especially coronary artery bypass graft (*P* = 0.046), a prolonged CPB time (*P* = 0.006), and a higher rate of intraoperative IABP installation (*P* = 0.02). Nevertheless, both the prosthetic type and size were similar between the groups. Table 2 illustrates that all of the factors described above were homogenized using propensity score matching, except the prevalence of DM (*P* = 0.005), serum creatinine level (*P* < 0.001), and eGFR (*P* < 0.001).

**TABLE 2:**
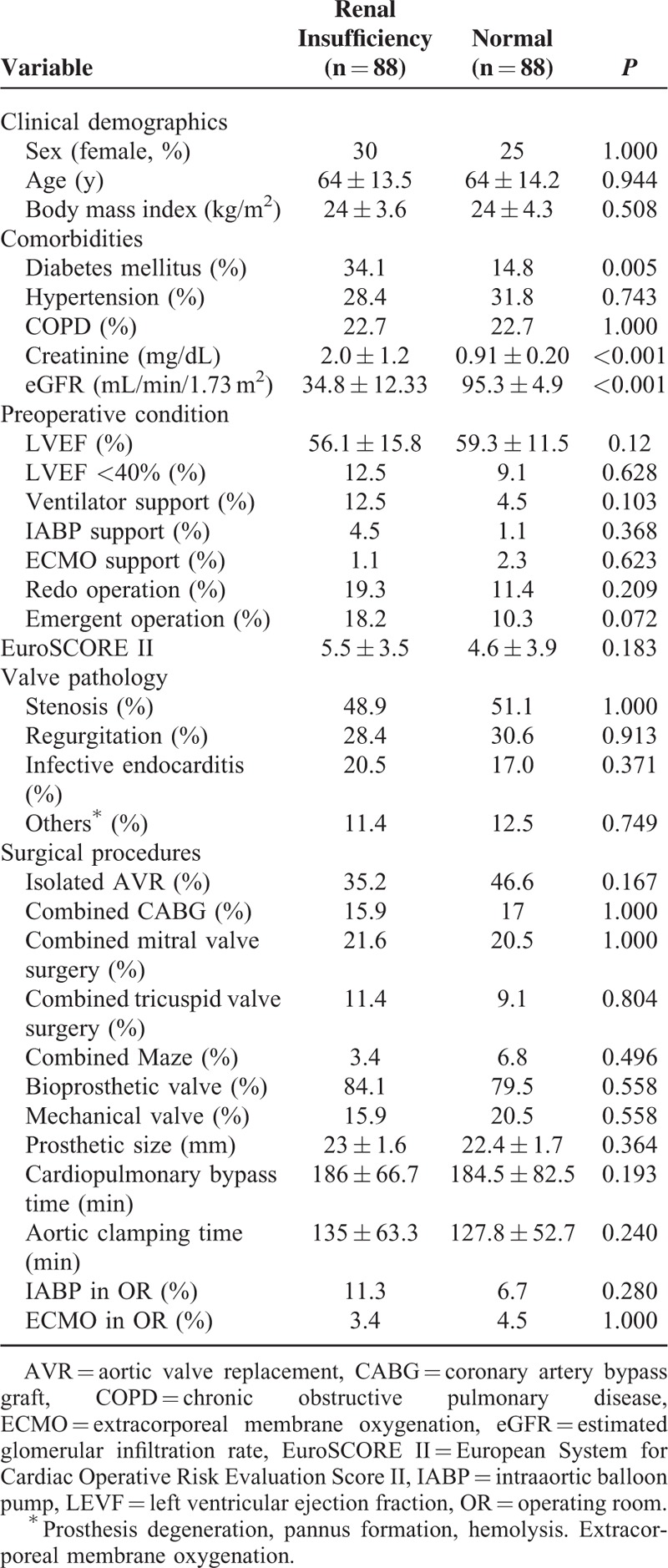
Comparisons of Propensity-matched Patients Between the Renal Insufficiency and Normal Groups

### Postoperative Mortality and Morbidity

Postoperatively, patients with renal insufficiency had significantly higher in-hospital mortality rates compared with normal patients (*P* < 0.001) (Table 3). In addition, postoperative complications, such as hemodialysis requirement (*P* = 0.009), prolonged ventilation support (*P* = 0.008), extended ICU stay (*P* = 0.046), and hospitalization (*P* = 0.008) were more prevalent in patients with renal insufficiency compared with normal patients. Patients with renal insufficiency also showed a high incidence of other complications, including acute kidney injury, check bleeding, atrial fibrillation, cerebral infarction, delirium, ICU readmission, and mechanical support system installation, but there was no statistical significance (Table 3).

**TABLE 3:**
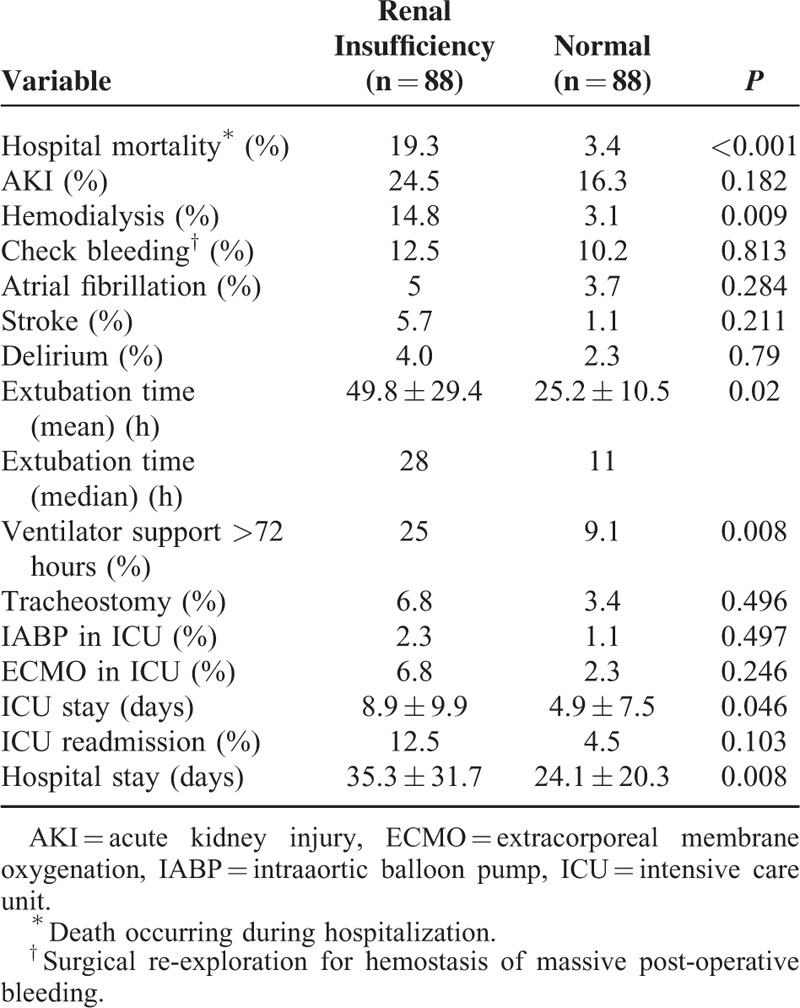
Comparisons of Postoperative Mortality and Morbidity

### Factors Associated with In-hospital Mortality

According to the multivariate analysis, preoperative renal insufficiency, IABP installation in the operating room, prolonged CPB time, and postoperative renal failure requiring hemodialysis were independent prognosticators of in-hospital mortality (Table 4). For the regression analysis of CPB time, the established model revealed good calibration (Hosmer–Lemeshow test; *P* = 0.72) and discrimination (area under receiver operating characteristic curve 0.751; *P* = 0.03).^9^

**TABLE 4:**
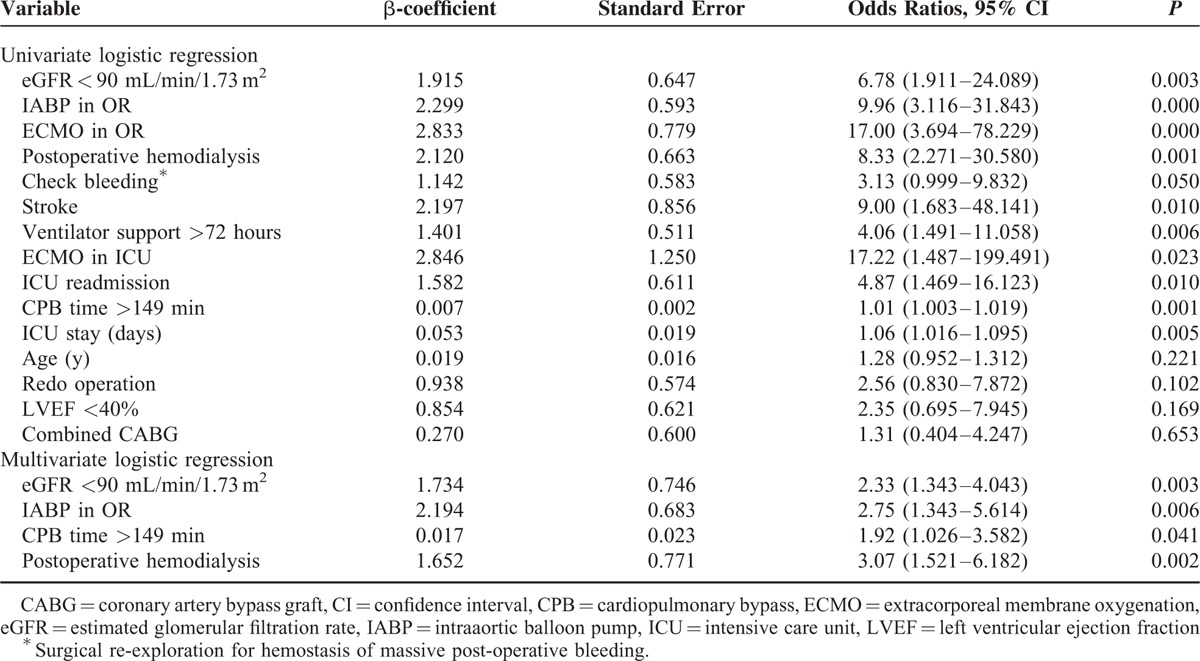
Prognostically Significant Variables for In-hospital Mortality

### Cumulative Postoperative Survival

**FIGURE 1:**
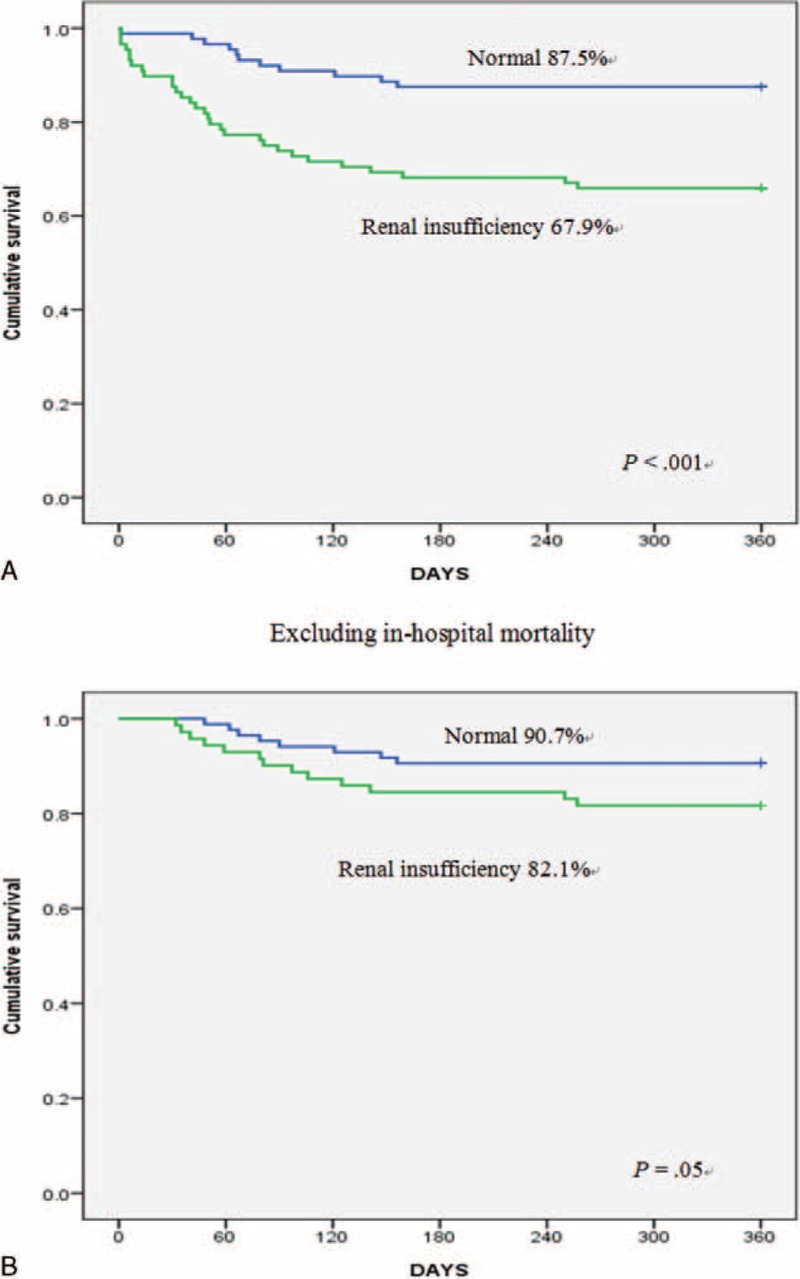
One-year cumulative survival rates after aortic valve replacement for 176 matched patients including in-hospital mortality (A) and for 155 patients excluding in-hospital mortality (B) based on preoperative renal function.

In the propensity-matched populations, the 1-year postoperative cumulative survival rates were significantly lower in patients with renal insufficiency compared with those in normal patients regardless of whether in-hospital mortality rates were included (normal, 87.5% versus renal insufficiency, 67.9%, *P* < .001) or not (normal, 90.7% versus renal insufficiency, 82.1%, *P* = .05; Figure 1).

## DISCUSSION

Patients with chronic kidney disease have a high prevalence of comorbidities, such as old age, hypertension, DM, chronic obstructive pulmonary disease, and deteriorated heart function.^10^ Therefore, the results of postoperative outcomes may be skewed by selection bias. No previous studies have applied propensity score analysis to evaluate outcomes in these patients. In the present study, propensity score analysis successfully minimized the bias of variables allowing meaningful comparisons between patients with and without renal insufficiency associated comorbidities. Despite this, the DM prevalence in the matched populations remained high in those with renal insufficiency. According to Katherine and Alan et al, diabetes has a major impact on the development of diabetic nephropathy, which is a common leading cause of chronic kidney disease or even ESRD.^11,12^ Therefore, it may be difficult to homogenize the disparity of DM prevalence in this study. However, according to the multivariate analysis, DM prevalence was not an independent predictor for in-hospital mortality.

As mentioned earlier, preoperative ESRD can increase surgical mortality rate and postoperative complication rate following cardiac surgery,^13^ including valvular operations.^14^ Several mechanisms, including metabolic instability, infection due to immune system compromise, ventilator dependence, bleeding tendency, or hemodynamic fluctuation under hemodialysis have been suggested to underlie these dismal outcomes.^15,16^ The present study found similar adverse effects, including significantly higher rates of in-hospital mortality, postoperative hemodialysis, respiratory failure with ventilator dependence, prolonged ICU, and hospitalization in patients with renal insufficiency compared with those without renal insufficiency.

According to the multivariate analysis, renal insufficiency itself was a strong independent predictor of in-hospital mortality, as were IABP installation in the operating room, prolonged CPB time, and postoperative renal failure requiring hemodialysis. Furthermore, patients with renal insufficiency were more likely to need complex surgical procedures, especially combined coronary artery bypass graft. Unsurprisingly, patients with preexisting ischemic-cardiomyopathies needing combined surgical procedures would inevitably exhibit prolonged CPB and aortic clamping times, which would contribute to intraoperative myocardial failure requiring mechanical support. Actually, patients with renal insufficiency had a higher DM prevalence rate compared with normal patients. Except the major impact to induce chronic kidney disease, DM is also a known risk factor for coronary artery disease.^17^ Furthermore, as Bove et al described, an IABP device can increase the risk of acute renal failure and induce complications after cardiac surgery.^18^ Moreover, patients with renal insufficiency had lower preoperative left ventricle ejection fractions and higher European System for Cardiac Operative Risk Evaluation Score II values, which have been implicated in both intraoperative myocardial failure and diminished survival. According to Zhibing et al, a low left ventricular ejection fraction was an independent predictor of in-hospital mortality in dialysis patients undergoing valvular surgery.^19^

Cardiopulmonary bypass conducted during cardiac surgery can incite a strong systemic inflammatory response that has been correlated with acute kidney injury (AKI).^20^ The proinflammatory triggers included intraoperative tissue trauma, blood component contact with the artificial surfaces of the CPB circuits, and reperfusion injury. Furthermore, renal cellular ischemia induced by redistributed organ perfusion during hypotensive operative processes may result in considerable tubular epithelium and vascular endothelium injury.^21^ Several studies have suggested that postoperative AKI would encumber surgical outcome and increase in-hospital mortality rates.^22,23^ Congruent with these suggestions, in the present study, patients with renal insufficiency had significantly longer CPB times (213.9 ± 107.5 minute versus 157 ± 78.6 minute, *P* = 0.006; Table 1) owing to more complex surgical procedures, and the CPB time was prolonged, even after propensity matching; both are much longer than the average CPB duration for isolated AVR cases (59–85 minutes) reported in previous studies.^24,25^ In addition, Boldt et al reported that patients with a prolonged CPB duration (>90 minutes) had more pronounced kidney damage and reduced postoperative renal function compared with those with shorter CPB durations (<70 min).^26^ Furthermore, such dismal effects may be magnified if patients have severe AKI requiring hemodialysis.

Postoperative hemodialysis was also a significant independent predictor of in-hospital mortality in the present study. There was also an interesting observation in the present study. Our study revealed a trend of more prevalent AKI in patients with renal insufficiency compared to those without renal insufficiency. However, the ratio of postoperative hemodialysis requirement was significantly different between the groups. These data can be explained via several reasons. First, patients in the normal group may endure AKI with less progression to acute renal failure and hemodialysis because of better-preserved preoperative renal function. In other words, preoperative renal dysfunction would elevate the risk of postoperative acute renal failure if AKI occurred. Furthermore, patients with comorbidities tend to have impaired auto-regulation of organ perfusion.^27^ Here, patients with renal insufficiency had a significantly high prevalence of those underlying comorbidities. Consequently, it could be hypothesized that multiple comorbidities or restricted preservation of renal function in patients with renal insufficiency induced the considerably high ratio of postoperative hemodialysis in the present study.

Despite the promising results of this study, several important limitations must be considered. First, the study was a retrospective and nonrandomized control trial with limited case numbers, suggesting that bias might exist regarding the homogeneity of the renal insufficiency and normal groups. However, by propensity matching, the heterogeneity was minimized, except for DM prevalence. Second, almost two-thirds of the patients underwent combined surgical procedures. Therefore, the mean CPB and aortic clamping times in each group were much longer than those in previous studies, which might affect the final outcomes. Besides, the MDRD equation has been developed for a Caucasian population and is not considered completely reliable for other races, even though an adaptation has been introduced for Chinese patients.^28^ Further, this equation may be also not reliable for patients over 70 years of age. Finally, as a retrospective study, hemodynamic profiles, such as changes in central venous pressure, pulmonary artery or wedge pressure, cardiac output, and inotropic medication dosage were not analyzed completely because of missing or incomplete records. This might hinder more detailed analyses of postoperative hemodynamic fluctuation.

In summary, patients with preoperative renal insufficiency who underwent AVR usually had higher comorbidity rates, poorer general condition, and required complex surgical procedures. Compared with those without renal insufficiency, they had higher in-hospital mortality rates and a greater risk of postoperative complications. Among these patients, the in-hospital mortality rate will rise remarkably if hemodynamic compromised with IABP installation or postoperative renal failure occurred. For patients with complicated comorbidities, we suggest that an earlier surgical intervention policy for severe aortic valve disease should be considered if renal function deteriorates during clinical follow-up.
